# Lessons from the Cerebrospinal Fluid Analysis of HTLV-1-Infected Individuals: Biomarkers of Inflammation for HAM/TSP Development

**DOI:** 10.3390/v14102146

**Published:** 2022-09-29

**Authors:** Nicole Lardini Freitas, Yago Côrtes Pinheiro Gomes, Flávia dos Santos Souza, Rafael Carvalho Torres, Juliana Echevarria-Lima, Ana Claudia Celestino Bezerra Leite, Marco Antonio Sales Dantas Lima, Abelardo Queiroz Campos Araújo, Marcus Tulius Teixeira Silva, Otávio de Melo Espíndola

**Affiliations:** 1 Instituto Nacional de Infectologia Evandro Chagas (INI), Fundação Oswaldo Cruz (FIOCRUZ), Rio de Janeiro 21040-900, Brazil; 2 Instituto Oswaldo Cruz (IOC), Fundação Oswaldo Cruz (FIOCRUZ), Rio de Janeiro 21040-900, Brazil; 3 Instituto de Biofísica Carlos Chagas Filho (IBCCF), Universidade Federal do Rio de Janeiro (UFRJ), Rio de Janeiro 21941-902, Brazil; 4 Instituto de Puericultura e Pediatria Martagão Gesteira, Universidade Federal do Rio de Janeiro (UFRJ), Rio de Janeiro 21941-912, Brazil; 5 Instituto de Microbiologia Paulo de Góes, Universidade Federal do Rio de Janeiro (UFRJ), Rio de Janeiro 21941-902, Brazil

**Keywords:** HTLV-1, HAM/TSP, biomarkers, cerebrospinal fluid, neurodegeneration, inflammasome, IL-18

## Abstract

HTLV-1-associated myelopathy/tropical spastic paraparesis (HAM/TSP) is a neurodegenerative disease that leads to motor impairment due to a chronic inflammatory process in the central nervous system (CNS). However, the HAM/TSP pathogenesis is not completely clear, and biomarkers to define the disease prognosis are still necessary. Thus, we aimed to identify biomarkers for HAM/TSP and potential mechanisms involved in disease development. To that end, the concentrations of VILIP-1, BDNF, VEGF, β-NGF, TGF-β1, fractalkine/CX3CL1, IL-6, IL-18, and TNF-α, and the soluble forms of TREM-1, TREM-2, and RAGE, were assessed using a multiplex bead-based immunoassay in paired cerebrospinal fluid (CSF) and serum samples from HAM/TSP patients (*n* = 20), asymptomatic HTLV-1 carriers (AC) (*n* = 13), and HTLV-1-seronegative individuals (*n* = 9), with the results analyzed according to the speed of HAM/TSP progression. HAM/TSP patients had elevated fractalkine in the serum but not in the CSF, particularly those with low neuroinflammatory activity (CSF/serum ratio of neopterin <1 and of CXCL10 < 2). HAM/TSP patients with normal CSF levels of neurofilament light chain (NfL) showed elevated β-NGF in serum, and serum BDNF levels were increased in HTLV-1-infected individuals, particularly in HTLV-1 AC. Both HTLV-1 AC and HAM/TSP patients had lower TGF-β1 levels in CSF compared to uninfected individuals, and HAM/TSP patients with active CNS inflammation showed higher CSF levels of IL-18, which correlated with markers of inflammation, neuronal death, and blood–brain-barrier permeability. Although none of the factors evaluated were associated with the speed of HAM/TSP progression, reduced TGF-β1 levels in CSF suggest that suppressive responses to control subclinical and/or active neurodegeneration are impaired, while increased CSF IL-18 indicates the involvement of inflammasome-mediated mechanisms in HAM/TSP development.

## 1. Introduction

The human T-lymphotropic virus 1 (HTLV-1) was the first human retrovirus described, in 1980 [1]. Currently, epidemiological studies indicate that HTLV-1 infection is endemic in Japan, the Caribbean, West and East Africa, Australia, the Middle East, and South America, particularly in Brazil, and it is estimated that from 5 to 10 million people are living with HTLV-1 worldwide [2]. HTLV-1 infection is associated with two major diseases: a malignancy of mature CD4^+^ T-cells named adult T-cell leukemia/lymphoma (ATLL), and HTLV-1-associated myelopathy/tropical spastic paraparesis (HAM/TSP), with 1–5% of infected individuals developing related diseases [3].

HAM/TSP is an inflammatory disorder affecting the central nervous system (CNS), in which demyelination and neuronal death are observed, leading to a progressive loss of motor function [4]. This neurodegenerative disease mainly affects the thoracic segment of the spinal cord, and most patients present with weakness in the lower limbs and consecutive falls as initial symptoms [3,5,6]. The disease can be also associated with lumbar pain, urinary disturbance, bowel constipation, erectile dysfunction, paresthesia, and neuropathic pain [3,4,7]. However, the mechanisms associated with the HAM/TSP pathogenesis are still not fully understood.

The most accepted hypothesis for HAM/TSP development is that neuronal damage results from CNS inflammation triggered by cytokines secreted by the infiltrate of infected CD4^+^ T-cells, particularly interferon-γ (IFN-γ) and tumor necrosis factor α (TNF-α) [3,4,5]. HTLV-1 infection modulates the function of CD4^+^ T-cells, which show an increased capacity for cytokine release, proliferation, and tissue migration [7,8,9]. In addition to elevated IFN-γ and TNF-α, the cerebrospinal fluid (CSF) of HAM/TSP patients shows elevated interleukin (IL)-1β, IL-2, IL-6, and granulocyte/macrophage-colony stimulating (GM-CSF) levels [8]. Histopathological analysis also showed that cellular infiltrates in active lesions in the spinal cord are mostly constituted of CD4^+^ T-cells and macrophages, and viral expression is also present, as shown by the detection of mRNAs coding for HTLV-1 Tax protein. In contrast, at later stages of the disease, lesions are characterized by infiltrating CD8^+^ cytotoxic T-cells (CTLs) and low viral expression [7]. Thus, inflammation associated with the antiviral response of HTLV-1-specific CTLs against infected cells expressing viral antigens promotes collateral neuronal damage [4,7]. In addition, higher CSF levels of Th1-related chemokines CXCL9 and CXCL10 are also present; they strongly correlate with the HTLV-1 proviral load (PVL) in peripheral blood mononuclear cells (PBMCs) and with the speed of HAM/TSP progression [10,11]. Indeed, CXCL10 has been demonstrated as a central mediator of the HAM/TSP pathogenesis by recruiting inflammatory and infected cells into the CNS [12]. Then, after months or years of neuroinflammation, irreversible macroscopic damage in the spinal cord can be evidenced by magnetic resonance imaging [4,13].

The HAM/TSP pathogenesis is a multifaceted process, and factors triggering disease development or determining the speed of disease progression are still under investigation. Previous observations have shown that biomarkers of inflammation provide stronger evidence of HAM/TSP progression than markers of neuronal injury, such as neurofilament and Tau proteins [11]. Moreover, HAM/TSP patients have elevated CSF levels of inflammatory chemokines, including CCL2, CCL3, CCL4, CCL17, CXCL5, CXCL10, and CXCL11, in addition to a higher frequency of Tax^+^CD4^+^ T-cells expressing CXCR3 in the peripheral blood [11]. The CNS has many mechanisms that are alert to sense injury, changes in homeostasis, and pathogen insult, and distinct cell types are involved in responses to infection and inflammation. Therefore, this study aimed to analyze CSF and serum samples from asymptomatic HTLV-1 carriers (AC) and HAM/TSP patients to identify alterations in factors commonly associated with the development of CNS diseases, and their possible involvement in the HAM/TSP pathogenesis. This included cytokines such as IL-6, TNF-α, IL-18, tumor growth factor (TGF)-β1, and fractalkine/CX3CL1, growth factors associated with neuronal and vascular homeostasis, such as BDNF (brain-derived neurotrophic factor), β-NGF (nerve growth factor β), and VEGF (vascular endothelial growth factor), sensors of metabolic stress, including visinin-like protein 1 (VILIP-1) and soluble receptor for advanced glycation end-products (sRAGE), and biomarkers of myeloid cells’ activation, such as the soluble triggering receptor expressed on myeloid cells 1 (sTREM-1) and sTREM-2.

## 2. Materials and Methods

### 2.1. Study Design and Population

This cross-sectional study included HTLV-1-infected patients (*n* = 33) older than 18 years recruited between August 2017 and August 2018 from the cohort of the Instituto Nacional de Infectologia Evandro Chagas (INI) of Fundação Oswaldo Cruz (FIOCRUZ), Rio de Janeiro, Brazil. Patients were excluded from the study when: diagnosed with co-infection with other viruses (HIV, HBV, HCV, and HTLV-2); submitted to treatment with corticosteroids or other immunomodulating drugs in the year prior to lumbar puncture; diagnosed with ATLL, autoimmune diseases, or other chronic inflammatory disorders; diagnosed with other diseases that impair the motor function, such as Parkinson’s syndrome, rheumatoid arthritis, ankylosing spondylitis, and others; presenting decubitus bedsores in the six months prior to the date of sample collection. The study was approved by the institutional review board (protocol number 53518416.9.0000.5262, March 2016), and paired CSF and serum samples were collected after written informed consent was obtained. Samples were processed as previously described [11] and kept at −80 °C until use.

The patients were classified into groups of HTLV-1 AC (*n* = 13) and HAM/TSP patients (*n* = 20). Clinical diagnosis of HAM/TSP was performed following the World Health Organization guidelines [14]. HAM/TSP patients were also subdivided according to the rate of disease progression. Briefly, patients with HAM/TSP were evaluated with the IPEC-2 disability scale, and a disease progression index was obtained by the quotient between the scores and the time of disease (interval between disease onset and sample collection) in years. HAM/TSP progression was characterized as: very slow (*n* = 6) for a disease progression index ≤0.37 points/year, typical (*n* = 8) for index values between 0.38 and 1.44 points/year, and rapid (*n* = 6) for an index ≥1.45 points/year [11]. Data from paired CSF (*n* = 9) and serum samples (*n* = 5) obtained from HTLV-1-seronegative individuals with non-inflammatory and non-infectious neurological diseases (normobaric hydrocephalus) collected in another study (protocol number 30611720.6.0000.5262, April 2020) were used as negative controls.

CSF samples were previously evaluated for cell counts and total protein levels, in addition to distinct biomarkers of neuronal damage, including Tau, neurofilament light (NfL), and phosphorylated neurofilament heavy (pNfH) proteins. Both the CSF and serum samples were also previously assessed to determine the concentration of neopterin and inflammatory chemokines, including CCL2, CCL3, CCL4, CCL5, CCL11, CCL17, CCL20, CXCL1, CXCL5, CXCL8, CXCL9, CXCL10, and CXCL11 [11]. Data on HTLV-1 PVL in PBMCs were obtained from medical records.

### 2.2. Quantification of Factors Associated with Neuroinflammation

The concentrations of VILIP-1, BDNF, TGF-β1, VEGF, IL-6, sTREM-1, sTREM-2, β-NGF, IL-18, TNF-α, sRAGE, and fractalkine/CX3CL1 in CSF and serum samples were simultaneously determined using a multiplex bead-based immunoassay (LEGENDPlex^TM^ Human Neuroinflammation Panel 1, Cat. No. 740795, Biolegend, USA). Serum samples were diluted at 1:2, following the manufacturer’s recommendations, and CSF samples were assessed undiluted. Data were collected by flow cytometry in a FACS Canto II (BD Biosciences, Franklin Lakes, NJ, USA), and the concentration of each factor was calculated in five-parameter logarithmic curves with the supplier’s software.

### 2.3. Statistical Analysis

Statistical analysis and graph elaboration were performed with *R* software v3.5.1. A normal distribution of quantitative variables was evaluated by the Kolmogorov–Smirnov test. Parametric variables were evaluated by Student’s *t*-test with Welch’s correction for distinct variances and Pearson’s correlation analysis. The analysis of parametric data was carried out with an ANOVA test with Bonferroni correction for multiple comparisons. Pairwise comparisons of non-parametric data were performed by a Mann–Whitney test and correlation analysis was carried out with Spearman’s rank correlation test. Non-parametric data analysis was performed with the Kruskal–Wallis test and Dunn’s post hoc test with correction for multiple comparisons. Associations between qualitative variables were defined by the chi-squared test, or Fisher’s exact test where indicated. Data from HTLV-1 AC and HAM/TSP patients were also analyzed with individuals subdivided as: (i) normal or altered CSF levels of NfL, considering reference values corrected for their age (18–29 years: <380 pg/mL; 30–39 years: <560 pg/mL; 40–58 years: <890 pg/mL; >59 years: <1850 pg/mL) [15]; (ii) normal CSF cell counts (<5 cells/mm^3^) or pleocytosis (≥5 cells/mm^3^); (iii) a CSF/serum ratio of neopterin <1 or ≥1; (iv) a CSF/serum CXCL10 ratio <2 or ≥2. Comparison between groups was performed with Wilcoxon’s test. Results with a *p*-value < 0.05 were considered significant.

## 3. Results

### 3.1. Clinical and Laboratory Aspects of the Study Population

The study population showed a similar distribution of individuals between groups of HAM/TSP patients, HTLV-1 AC, and HTLV-1-seronegative controls according to sex (chi-squared test, *p =* 0.935) and age (ANOVA, *p* = 0.345) (Table 1). Most HAM/TSP patients had chronic disease, with a mean time of disease of 12.94 ± 8.07 years, and 65% (*n* = 13) had a low level of motor disability, while 25% (*n* = 5) showed moderate impairment, and 10% (*n* = 2) displayed severe HAM/TSP disability (Table 1). As expected, HAM/TSP patients showed higher mean HTLV-1 PVL in PBMCs (8.46% ± 6.37% of infected cells/PBMCs) than HTLV-1 ACs (4.50% ± 3.64% of infected cells/PBMCs) (Student’s *t*-test *p* = 0.049) (Table 1).

All CSF samples had insignificant contamination with peripheral blood during the lumbar puncture, as evidenced by a median of 1 red blood cell (RBC)/mm^3^ (interquartile range: 0–6 RBC/mm^3^). The median total protein levels in the CSF were slightly elevated in HTLV-1 AC (Kruskal–Wallis, *p* = 0.020), and 53.8% of the patients in the group had levels higher than the reference values (15–45 mg/dL) (Table 1). In turn, altered total protein levels were observed in 30% of HAM/TSP patients, and all HTLV-1-seronegative donors in the control group showed normal levels. Although the frequency of patients with increased CSF total proteins was higher among HTLV-1 AC (chi-squared, *p* = 0.027), this finding was considered clinically irrelevant. In addition, the CSF of all participants presented 100% mononuclear cells, and all individuals in the HTLV-1 AC and control groups showed normal CSF cell counts (<5 cells/mm^3^). Although eight HAM/TSP patients had pleocytosis, the median CSF cell counts were within the normal range (4 cells/mm^3^, IQR 1.5–7.5 cells/mm^3^), though significantly higher compared to the HTLV-1 AC and control groups (Kruskal–Wallis, *p* < 0.001) (Table 1).

### 3.2. Analysis of Inflammatory Factors in the Serum of HTLV-1-Infected Individuals

**Figure 1:**
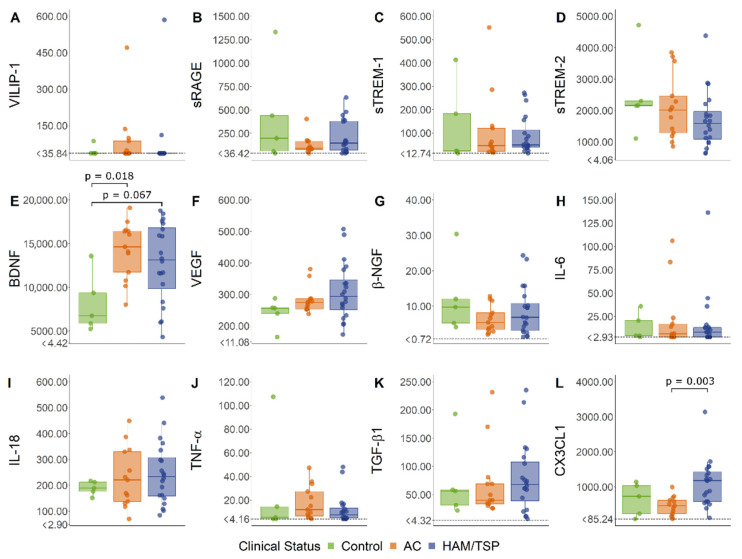
Serum levels of common biomarkers of neuroinflammation. (**A**) VILIP-1, (**B**) sRAGE, (**C**) sTREM-1, (**D**) sTREM-2, (**E**) BDNF, (**F**) VEGF, (**G**) β-NGF, (**H**) IL-6, (**I**) IL-18, (**J**) TNF-α, (**K**) TGF-β1, and (**L**) CX3CL1 (fractalkine) levels were simultaneously determined with a multiplex bead-based immunoassay by flow cytometry in serum samples from asymptomatic HTLV-1 carriers (AC) (*n* = 13), HAM/TSP patients (*n* = 20), and a control group of HTLV-1-seronegative individuals (*n* = 5). A normal distribution was determined by the Kolmogorov–Smirnov test. The statistical analysis of parametric variables was performed with ANOVA and Bonferroni’s post-test, and non-parametric data were evaluated by the Kruskal–Wallis test and Dunn’s post-test. Results with a *p*-value < 0.05 were considered significant.

The serum levels of VILIP-1, sRAGE, sTREM-1, sTREM-2, VEGF, β-NGF, IL-6, IL-18, TNF-α, and TGF-β1 did not show significant differences between HAM/TSP patients, HTLV-1 AC, and HTLV-1-seronegative controls (Figure 1). In contrast, the serum BDNF was increased in HTLV-1-infected individuals, particularly in HTLV-1 AC, in the comparison with control levels. In turn, serum fractalkine/CX3CL1 was elevated in HAM/TSP patients (Figure 1).

**Figure 2:**
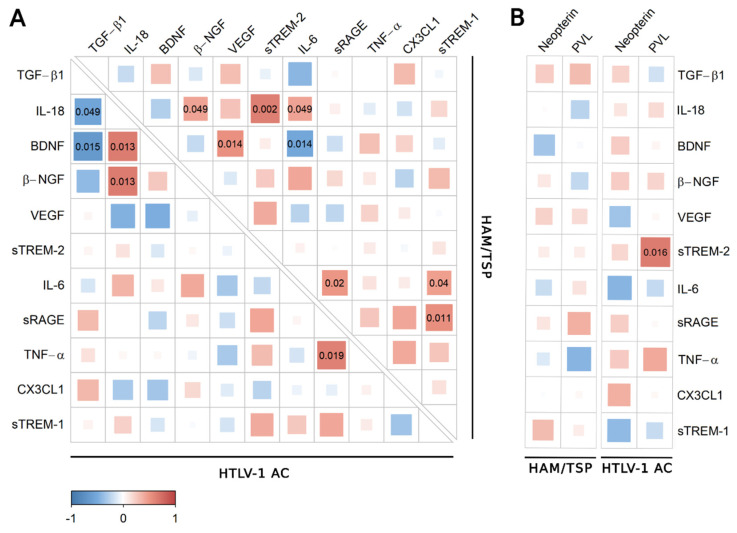
Correlation analysis of biomarkers of neuroinflammation in serum. (**A**) Correlation between TGF-β1, IL-18, BDNF, β-NGF, VEGF, sTREM-2, IL-6, sRAGE, TNF-α, CX3CL1 (Fractalkine), and sTREM-1 levels in serum samples from asymptomatic HTLV-1 carriers (AC) (*n* = 13) and HAM/TSP patients (*n* = 20) was performed with Spearman’s rank correlation test. (**B**) The analysis was also performed with the serum neopterin concentration and HTLV-1 proviral load (PVL) in PBMCs from HTLV-1 AC and HAM/TSP patients. Correlation coefficients are indicated by the color intensity, in which positive correlations are shown in red and negative correlations in blue. The size of the squares at intersections between factors represents the *p*-value, which is shown only for significant associations (*p* < 0.05).

Correlation analysis revealed distinct associations between factors in the serum of HTLV-1-infected individuals according to the neurological status (Figure 2). Since VILIP-1 was under the LLOD in more than 50% of serum samples of the study population, this factor was excluded from the analysis. In HTLV-1 AC, IL-18 levels increased with BDNF and β-NGF, while TNF-α was positively correlated with sRAGE (Figure 2A). In contrast, serum BDNF and IL-18 levels were negatively correlated with TGF-β1, a cytokine with anti-inflammatory properties (Figure 2A). In HAM/TSP patients, IL-18 increased with β-NGF, sTREM-2, and IL-6. The levels of IL-6, sTREM-1, and sRAGE were positively correlated, while BDNF levels showed a positive association with VEGF and a negative correlation with IL-6 (Figure 2A). Serum levels of none of these factors were associated with HTLV-1 PVL (Figure 2B), and serum neopterin was associated with sTREM-2 in HTLV-1 AC (Figure 2B).

**Figure 3:**
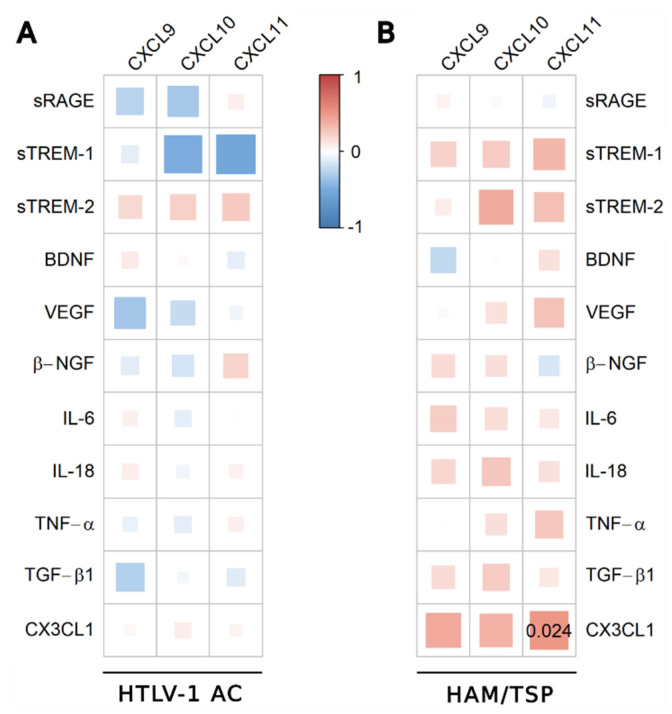
Correlation analysis between serum levels of inflammatory chemokines and factors associated with neuroinflammation. Correlation between serum levels of TGF-β1, IL-18, BDNF, β-NGF, VEGF, sTREM-2, IL-6, sRAGE, TNF-α, CX3CL1 (fractalkine), sTREM-1, and chemokines (CXCL9, CXCL10, and CXCL11) was evaluated in (**A**) asymptomatic HTLV-1 carriers (AC) (*n* = 13) and (**B**) HAM/TSP patients (*n* = 20) using the Spearman’s rank correlation test. Correlation coefficients are indicated by color intensity, in which positive correlations are shown in red and negative correlations in blue. The size of the squares at intersections between factors represents the *p*-value, which is shown only for significant associations (*p* < 0.05).

The analysis was also conducted using the serum concentration of the inflammatory chemokines CXCL9, CXCL10, and CXCL11, which were significantly increased in HAM/TSP patients, as previously described [11]. In HTLV-1 AC, only fractalkine/CX3CL1 was negatively correlated with CXCL11 (Figure 3A), and no correlation was observed among HAM/TSP patients (Figure 3B).

### 3.3. CSF Analysis of Biomarkers of Inflammation

**Figure 4:**
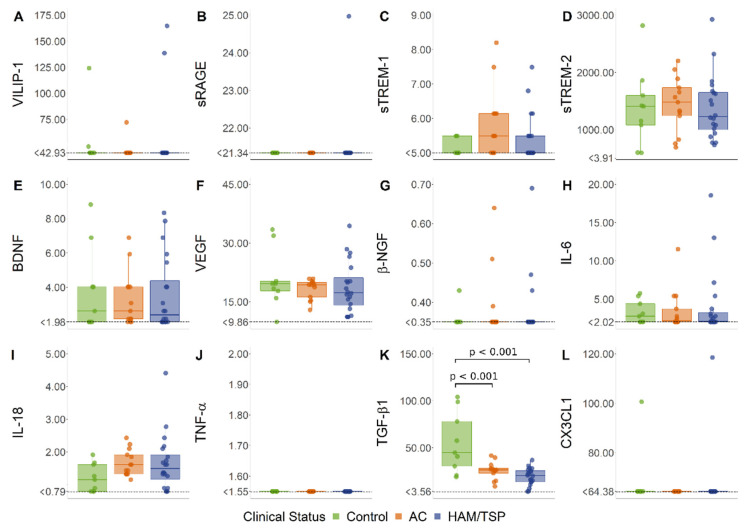
CSF levels of factors associated with neuroinflammation. (**A**) VILIP-1, (**B**) sRAGE, (**C**) sTREM-1, (**D**) sTREM-2, (**E**) BDNF, (**F**) VEGF, (**G**) β-NGF, (**H**) IL-6, (**I**) IL-18, (**J**) TNF-α, (**K**) TGF-β1, and (**L**) fractalkine (CX3CL1) levels were simultaneously determined with a multiplex bead-based immunoassay by flow cytometry in cerebrospinal fluid (CSF) samples from asymptomatic HTLV-1 carriers (AC) (*n* = 13), HAM/TSP patients (*n* = 20), and a control group of HTLV-1-seronegative individuals (*n* = 9). A normal distribution was determined by the Kolmogorov–Smirnov test. Statistical analysis of parametric variables was performed with ANOVA and Bonferroni’s post-test, and non-parametric data were evaluated by the Kruskal–Wallis test and Dunn’s post-test. Results with a *p*-value < 0.05 were considered significant.

**Figure 5:**
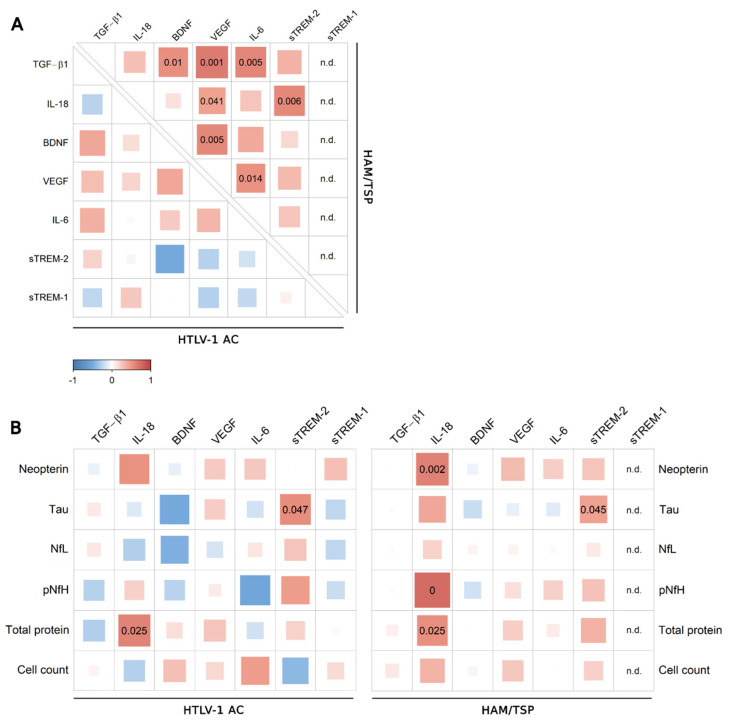
Correlation analysis of CSF levels of factors associated with neuroinflammation. (**A**) Correlation between CSF levels of TGF-β1, IL-18, BDNF, VEGF, IL-6, sTREM-2, and sTREM-1 in samples from asymptomatic HTLV-1 carriers (AC) (*n* = 13) and HAM/TSP patients (*n* = 20) was performed with Spearman’s rank correlation test. (**B**) Correlation analysis was also performed with the CSF levels of neopterin, Tau protein, neurofilament light chain (NfL) and phosphorylated neurofilament heavy chain (pNfH) proteins, total proteins, and CSF cell counts from HTLV-1 AC and HAM/TSP patients. Correlation coefficients are indicated by color intensity, in which positive correlations are shown in red and negative correlations in blue. The size of the squares at intersections between factors represents the *p*-value, which is shown only for significant associations (*p* < 0.05).

HTLV-1 AC and HAM/TSP patients displayed similar CSF levels of VILIP-1, sRAGE, sTREM-1, sTREM-2, BDNF, VEGF, β-NGF, IL-6, IL-18, TNF-α, and fractalkine/CX3CL1 (Figure 4). In contrast, TGF-β1 was reduced in both HTLV-1 AC and HAM/TSP patients in comparison with HTLV-1-seronegative controls (Figure 4). VILIP-1, sRAGE, β-NGF, TNF-α, and fractalkine/CX3CL1 were under the LLOD in more than 50% of the samples and, therefore, were excluded from further analysis. In HTLV-1 AC, no association was observed between neuroinflammatory factors (Figure 5A). In contrast, a high frequency of associations was present in HAM/TSP patients, particularly between TGF-β1, IL-6, IL-18, VEGF, and BDNF (Figure 5A).

**Figure 6:**
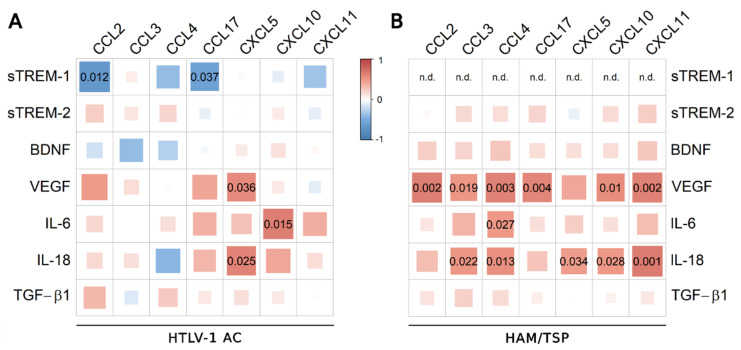
Correlation between CSF levels of inflammatory chemokines and factors associated with neuroinflammation. Correlation between CSF levels of TGF-β1, IL-18, BDNF, VEGF, IL-6, sTREM-2, sTREM-1, and chemokines (CCL2, CCL3, CCL4, CCL17, CXCL5, CXCL10, and CXCL11) was evaluated in (**A**) asymptomatic HTLV-1 carriers (AC) (*n* = 13) and (**B**) HAM/TSP patients (*n* = 20) with Spearman’s rank correlation test. Correlation coefficients are indicated by color intensity, in which positive correlations are shown in red and negative correlations in blue. The size of the squares at intersections between factors represents the *p*-value, which is shown only for significant associations (*p* < 0.05).

Correlation analysis was performed including CSF findings for the total proteins and cell counts, and the CSF concentrations of neopterin, chemokines, and biomarkers of neuronal damage (Tau protein, NfL, and pNfH). Positive correlations were observed between sTREM-2 and Tau protein and between IL-18 and total proteins in the CSF of HTLV-1 AC and HAM/TSP patients (Figure 5B). In turn, CSF IL-18 positively correlated with neopterin, pNfH, and total proteins only in HAM/TSP patients (Figure 5B). Scarce associations between neuroinflammatory factors and chemokines were observed in HTLV-1 AC (Figure 6A). Meanwhile, in HAM/TSP patients, VEGF and IL-18 levels were consistently correlated with many inflammatory chemokines that were significantly increased in the CSF of these individuals, as previously described [11] (Figure 6B), including CCL2, CCL3, CCL4, CCL17, CXCL5, CXCL10, and CXCL11.

**Figure 7:**
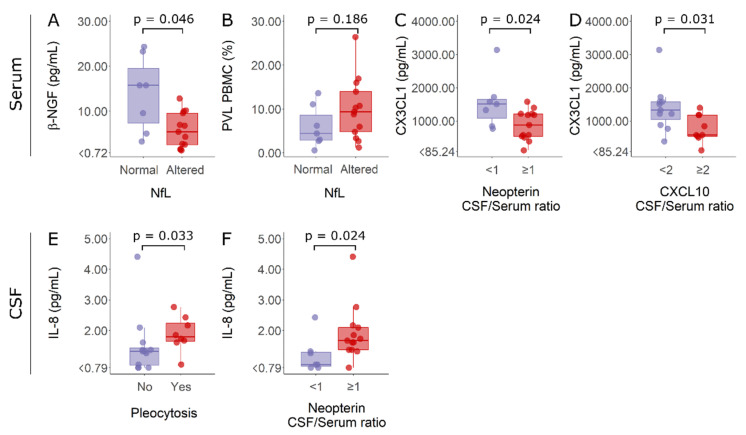
Serum β-NGF and fractalkine/CX3CL1 levels, HTLV-1 proviral load, and CSF IL-18 concentration in HAM/TSP patients according to altered markers of inflammation and neurodegeneration. Individuals were separated according to normal (*n* = 7, purple) and altered (*n* = 13, red) CSF levels of neurofilament light (NfL) proteins and considering active neuroinflammation as a CSF/serum ratio of neopterin ≥1 (*n* = 13, red) or CXCL10 ≥ 2 (*n* = 9, red). Patients with a CSF/serum ratio of neopterin <1 (*n* = 7, purple) or CXCL10 < 2 (*n* = 11, purple) were considered to have low neuroinflammatory activity. Finally, individuals were also discriminated by normal CSF cell counts (<5 cells/mm^3^) (*n* = 12, purple) or pleocytosis (*n* = 8, red). (**A**) Serum β-NGF, (**B**) HTLV-1 proviral load (PVL), and (**C**,**D**) serum fractalkine/CX3CL1 levels were compared between groups with Student’s *t*-test. (**E**,**F**) CSF IL-18 levels were compared between groups with the Mann–Whitney test. Differences with *p* < 0.05 were considered significant.

Serum and CSF data on factors associated with inflammatory processes were also evaluated according to alterations in biomarkers of inflammation and neurodegeneration. In this way, HAM/TSP patients were categorized according to whether they had normal CSF cell counts or pleocytosis, normal or altered CSF NfL, and active neuroinflammation or low neuroinflammatory activity, which was indicated by the CSF/serum ratios of neopterin and CXCL10. It was observed that HAM/TSP patients with normal NfL levels in the CSF showed higher serum β-NGF than patients with elevated NfL (Figure 7A). No difference, however, was observed in HTLV-1 PVL for HAM/TSP patients according to their NfL levels (Figure 7B). In turn, HAM/TSP patients with low neuroinflammatory activity presented higher serum levels of fractalkine/CX3CL1 (Figure 7C,D). Moreover, HAM/TSP patients with pleocytosis or active neuroinflammation, which was indicated by CSF/serum neopterin ≥1, showed higher IL-18 levels in the CSF (Figure 7E,F, respectively).

**Figure 8:**
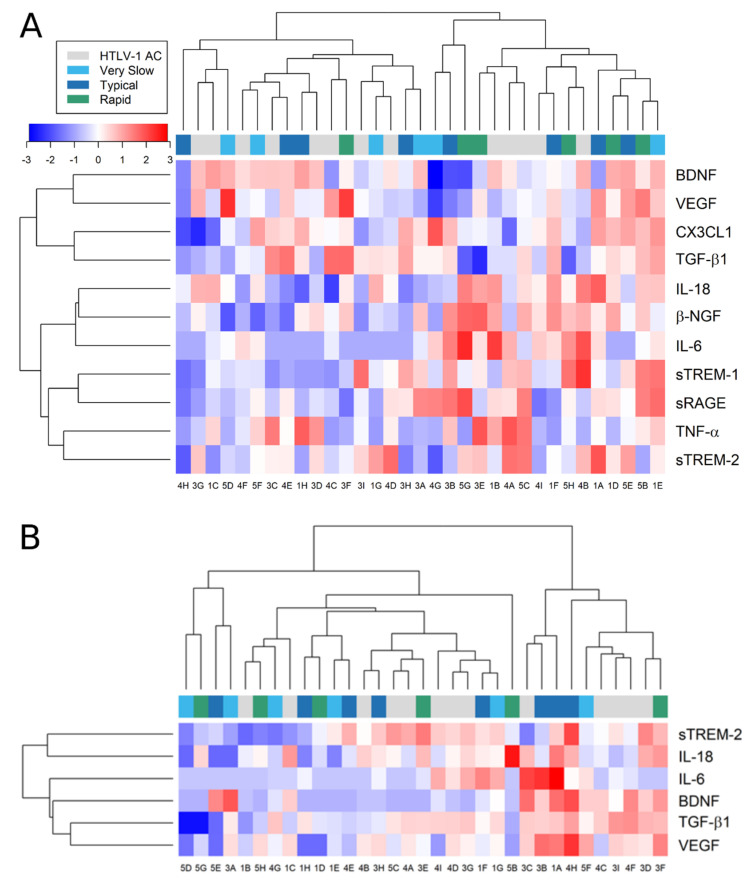
Relative expression of factors associated with neuroinflammatory processes. The concentration of factors listed was determined in the (**A**) serum and (**B**) CSF of asymptomatic HTLV-1 carriers (AC) (*n* = 13) and HAM/TSP patients (*n* = 20), which were further characterized by the speed of disease progression as very slow (light blue), typical (dark blue), or rapid (green), as previously described [11]. Only factors with detectable levels in more than half of the samples were included in the analysis. Values were log_2_-transformed for heatmap analysis, and the expression normalized for each factor (lines). Reduced expression is represented by shades of blue, increased expression by shades of red, and the median is indicated by 0 (white).

Relative expression analysis was also performed to identify inflammatory profiles associated with HTLV-1 AC and HAM/TSP patients (Figure 8). First, HAM/TSP patients were subdivided according to the speed of disease progression (very slow, typical, or rapid), as previously described [11]. Afterward, the levels of factors investigated were normalized for each factor, and clustering analysis was carried out to gather individuals with similar characteristics. However, the analysis resulted in a mixed distribution of HTLV-1 AC and HAM/TSP patients, reflecting the high diversity of profiles observed both in the serum (Figure 8A) and in the CSF (Figure 8B). This indicated that no specific combination of factors was associated with the asymptomatic condition or HAM/TSP.

## 4. Discussion

HTLV-1 infection is associated with HAM/TSP development in 1–5% of individuals after a long period of apparently asymptomatic infection. Thus, the identification of biomarkers to predict disease development and/or progression has become an important research topic since laboratory markers to support the clinical follow-up of HTLV-1 carriers are still under investigation.

Reduced TGF-β1 levels were observed in the CSF of HTLV-1-infected individuals compared to the HTLV-1-seronegative group, independently of the neurological status. TGF-β1 is a key factor in the modulation of cerebral damage, as well as exerting neuroprotective functions, and it is found to be reduced in neurodegenerative diseases [16]. Brain magnetic resonance imaging of HTLV-1-infected individuals has shown that both HAM/TSP patients and HTLV-1 AC present white matter lesions, indicating that the latter might display subclinical neurodegenerative conditions [17]. In HAM/TSP patients, CD4^+^ T-cells show some degree of resistance to anti-proliferative effects from TGF-β signaling, which is known to suppress the expansion of activated T-cells and modulate T-cell differentiation [18,19,20,21]. In the CNS, TGF-β participates in the cross-talk between astrocytes and microglia to maintain brain homeostasis. Under inflammatory stimuli, microglia cells secrete IL-10, which induces astrocytes to produce TGF-β1, which, in turn, downregulates microglial proinflammatory responses. In Alzheimer’s disease, β-amyloid plaques are surrounded by reactive glial cells (microglia and astrocytes) showing elevated expression of proinflammatory factors such as IL-1β, IL-6, TNF-α, and nitric oxide, while anti-inflammatory cytokines (TGF-β1 and IL-10) are at reduced levels [22,23]. Although increased TNF-α and IL-6 levels were not evident in the CSF in our study population, reduced TGF-β1 in HTLV-1-infected individuals might be related to chronic inflammatory stimulation in the CNS by infected cells, which are present in the CSF of both HTLV-1 AC and HAM/TSP patients, as previously shown [11].

HAM/TSP patients showed increased serum fractalkine/CX3CL1 levels. Soluble fractalkine is generated by cleavage of its transmembrane form by metalloproteinases ADAM10 and ADAM17 [24]. Fractalkine is chemoattractive to NK cells, monocytes, and T-cells, and it is expressed by endothelial cells, dendritic cells, and monocytes from peripheral blood under inflammatory conditions. Fractalkine expression is particularly induced by TNF-α and IFN-γ [24], which are cytokines elevated in HAM/TSP patients [25]. HAM/TSP patients also display higher expression of IFN-γ-stimulated genes in monocytes [26], and PBMCs from these patients secrete more IL-2 and IFN-γ than cells from HTLV-1 AC [27]. Indeed, it was shown that HAM/TSP patients have a higher frequency of T CD4^+^IFN-γ^+^ cells, which positively correlate with HTLV-1 PVL [28,29].

In turn, HTLV-1 AC presented higher serum BDNF levels. BDNF is a member of the neurotrophin family that acts on the survival, growth, and synaptic plasticity of neurons. In blood, BDNF is present in platelets, and it is produced by vascular endothelial cells and PBMCs. Moreover, coronary and lung cells also express BDNF [30,31]. In HTLV-1-infected cell lineages derived from ATLL patients, it was shown that BDNF expression is regulated by the HTLV-1 bZIP factor (HBZ) protein [32]. In PBMCs from HAM/TSP patients, *HBZ* mRNA expression correlates with HTLV-1 PVL, and no difference is observed between HTLV-1 AC and HAM/TSP patients [25]. However, serum BDNF and HTLV-1 PVL in PBMCs had no correlation. Therefore, elevated serum BDNF levels are likely influenced by factors other than *HBZ* expression in infected cells. However, more studies are needed to identify the mechanisms in addition to *HBZ* expression behind this event.

In the serum of HAM/TSP patients, BDNF levels were positively correlated with VEGF and negatively with IL-6. Serum IL-18 levels were positively correlated with IL-6, β-NGF, and sTREM-2. Although no differences were observed between groups, these associations were not present in HTLV-1 AC, except for IL-18 and β-NGF levels. Data from animal models have shown that BDNF and VEGF mediate physical exercise-induced structural and functional changes in the hippocampus. VEGF is able to promote the formation and growth of blood vessels, which improve cognitive function [33,34]. BDNF and VEGF can cross the BBB and mediate both neurogenesis and angiogenesis, which are related processes in the CNS since vascular development promotes a microenvironment that favors the proliferation and differentiation of neuronal progenitor cells [35,36,37]. Indeed, the recovery of patients after cerebrovascular accidents and traumatic cerebral lesions is associated with angiogenesis and neurogenesis [35]. ATLL and HAM/TSP patients display serum VEGF and b-FGF levels capable of inducing angiogenesis, which presents a difference from healthy HTLV-1-seronegative individuals [38]. The VEGF receptor is expressed by endothelial progenitors and monocytes, and it drives the recruitment of these cells [39]. HTLV-1-transformed cells also secrete VEGF and b-FGF in an HTLV-1 Tax protein-dependent process. Although this difference was not observed in our study population, activation of endothelial cells and altered vascular permeability triggered by infected cells may have a role in the HAM/TSP pathogenesis. Indeed, VEGF levels in the CSF of HAM/TSP patients were consistently correlated with distinct inflammatory cytokines, such as CCL2, CCL3, CCL4, CCL17, CXCL10, and CXCL11. These chemokines are related to the chemotaxis and migration of Th1 cells and monocytes, which supports the hypothesis that VEGF might contribute to alterations in BBB permeability and neuroinvasion by HTLV-1-infected cells.

In the CSF of HAM/TSP patients, IL-18 was correlated with sTREM-2, neopterin, pNfH, and the total protein concentration, suggesting an association between IL-18 levels and common biomarkers of inflammation and neurodegeneration. IL-18 is an inflammatory cytokine that induces IFN-γ production by Th1 cells. In non-polarized T cells, NK and NKT cells, B cells, dendritic cells, and macrophages, IFN-γ upregulation by IL-18 signaling is IL-12-dependent. In contrast, CD4^+^ T-cells, NK, and NKT cells exposed to IL-18 and IL-2 in the absence of IL-12 are induced to produce Th2 cytokines. Moreover, IL-18 associated with IL-3 induces IL-4 and IL-13 expression by mastocytes and basophils. Thus, IL-18 can stimulate distinct cellular types of innate and adaptative immunity considering the inflammatory setting [40,41]. sTREM-2 represents the cleaved form of TREM-2, a membrane receptor associated with DAP12 adaptor protein that suppresses lipopolysaccharide (LPS)-induced Toll-like receptor activation. TREM-2 is expressed by myeloid cells, including macrophages, monocyte-derived dendritic cells, and osteoclasts. In the SNC, TREM-2 is particularly expressed by microglia cells and participates in the regulation of cytokine production, phagocytosis of apoptotic neurons, and cell survival [42,43]. Since TREM-2 has an anti-inflammatory role, sTREM-2 negatively regulates TREM-2, thereby promoting inflammation and neurodegeneration [44]. It was shown that patients with the remitting-recurrent and progressive primary forms of multiple sclerosis have high CSF levels of sTREM-2 [45,46]. However, sTREM-2 was not elevated in the CSF of HAM/TSP patients, likely a result of the chronically slow progression of this disease. No association was observed either for sTREM-1, which has anti-inflammatory activity and mediates negative regulation of sTREM-2 [47].

IL-18 in the CSF also positively correlated with distinct proinflammatory chemokines in HAM/TSP patients, including CCL3, CCL4, CXCL5, CXCL10, and CXCL11. CXCL10 and CXCL11 are CXCR3 receptor ligands, and these chemokines are strongly induced in response to IFN-γ. In HTLV-1 infection, it has been shown that CXCL10 plays a central role in the recruitment of CXCR3^+^ cells to the CNS, for both infected CD4^+^ T-cells and IFN-γ^+^ secreting T-cells [12]. HAM/TSP patients show a higher frequency of Tax^+^CD4^+^ T-cells in the peripheral blood [11] and higher IFN-γ expression in PBMCs in comparison to HTLV-1 AC [25]. Thus, IFN-γ stimulates the secretion of CXCL10 by astrocytes, the main source of this chemokine in the CNS, resulting in positive regulatory feedback for the chronic CNS inflammation that characterizes HAM/TSP [4]. On the other hand, sTREM-2 and IL-18 data suggest that microglia cells might also be involved in triggering CNS inflammation in HTLV-1 infection.

IL-18 is a member of the IL-1 family, and it is present in myeloid cells as pro-IL-18 cytoplasmatic stocks [48]. The production of biologically active IL-18 depends on the cleavage of the pro-IL-18 inactive precursor by caspase-1. Thereafter, IL-18 is secreted or expressed as a membrane-bound ligand. In the CNS, both astrocytes and microglia constitutively express pro-IL-18 and caspase-1, leading to a local IL-18-dependent immune response [49]. It was shown that HTLV-1-infected individuals have reduced *IL18* gene expression and plasma levels compared to HTLV-1-seronegative individuals, but no difference was observed between HTLV-1 AC and HAM/TSP patients [50]. In our study, no change in the serum or CSF IL-18 concentration was observed among the groups. However, HAM/TSP patients with pleocytosis or active neuroinflammation had higher IL-18 levels in the CSF, suggesting that inflammasome activity is involved in the HAM/TSP pathogenesis. Inflammasomes are multiprotein complexes in the cytoplasm that activate proinflammatory caspases, which are responsible for the maturation and subsequent secretion of IL-1β and IL-18. Inflammasome activation is triggered by the ligation of pathogen- and/or damage-associated molecular patterns to receptors such as Toll-like receptor and NOD [51]. In the CNS, inflammasome signaling is mainly performed by microglia cells. However, neurons, astrocytes, perivascular macrophages, oligodendrocytes, and endothelial cells also express inflammasome components [52]. In multiple sclerosis, BBB disruption and the infiltration of inflammatory cells from the periphery are driven by the activation of microglia and astrocytes, leading to inflammation, demyelination, and consequent neurodegeneration. In multiple sclerosis, caspase-1, IL-18, and IL-1β are positively regulated in PBMCs and in mononuclear cells in the CSF during disease development, and caspase-1 expression is upregulated in demyelinating lesions [53,54]. Thus, it will be of particular importance to study the role of microglia cells and inflammasome activation in HAM/TSP pathogenesis.

Differences in CSF levels of β-NGF among HTLV-1 AC, HAM/TSP patients, and uninfected individuals were not evident, as also observed by Albrecht et al. [55]. However, detailed analysis showed that serum β-NGF concentrations were higher in HAM/TSP patients with normal age-adjusted NfL levels in the CSF. Although β-NGF is capable of inducing neurogenesis and increasing neuron survival and plasticity, this neurotrophin does not cross the BBB [56]. In inflammatory and autoimmune diseases, a localized increase in NGF is observed at sites of inflammation. Mast cells, lymphocytes, and eosinophils can produce, store, and secrete NGF, and NGF receptors are widely expressed by immune cells, thus mediating an autochthonous response [57,58,59,60]. Elevated NGF levels are present in the CSF of patients with multiple sclerosis [61], in the synovial fluid of patients with rheumatoid arthritis [62,63], and in the serum of individuals with systemic erythematosus lupus [64,65]. It is not clear whether peripheral responses involving β-NGF are directly associated with reduced neuronal death and/or neuroinflammation in HAM/TSP. One hypothesis could be that higher serum β-NGF in HAM/TSP patients is related to more effective control of inflammatory events leading to the development of neurological lesions, independently of the control of infected T-cell populations in the periphery, since the HTLV-1 PVL in PBMCs was similar between HAM/TSP patients with normal and elevated NfL. In the future, further studies are needed to explain this finding.

In HTLV-1-infected individuals, no change in sRAGE levels was observed. The interaction between RAGE and its ligands on the cell plasma membrane results in the formation of reactive oxygen species (ROS) that activate NF-kB [66], leading to the expression of proinflammatory genes [67]. Under normal conditions, RAGE is expressed at low levels [68]. However, with pathophysiological conditions, such as diabetes, chronic inflammation, or neurodegenerative disturbances, RAGE levels can be elevated [69,70,71]. In this regard, our data indicate that RAGE signaling is not altered by HTLV-1 infection.

HAM/TSP patients with low neuroinflammation activity, as indicated by the CSF/serum ratio of neopterin and CXCL10, had higher serum fractalkine/CX3CL1 levels compared to patients with active neuroinflammation. In peripheral blood, fractalkine/CX3CL1 is mainly expressed by monocytes under inflammatory conditions. Non-classic monocytes monitor the blood/endothelial barrier, and low CCR2 expression in these cells leads to reduced transendothelial migration ability, meaning they exert anti-inflammatory functions [72,73,74,75]. In turn, non-classic CD16^+^ monocytes mainly express CX3CR1, and, therefore, migrate in response to fractalkine/CX3CL1 [76]. De Castro-Amarante et al. have shown that the frequency of non-classic monocytes is increased in HTLV-1-infected individuals, and this cell population also shows increased expression levels of CCR1, CXCR3, and CX3CR1 chemokine receptors [77]. Thus, it was hypothesized that inflammation in HTLV-1 infection results from the regulation of chemokine receptors expressed in monocytes. In general, inflammatory responses favor the migration of classic monocytes, the most abundant monocyte population in peripheral blood, which mainly respond to CCL2 signaling. On the other hand, increased fractalkine/CX3CL1 levels in HAM/TSP patients, and particularly in those with low neuroinflammatory activity, corroborate the findings of increased anti-inflammatory non-classic monocyte populations.

In conclusion, VILIP-1, sRAGE, sTREM-1, sTREM-2, BDNF, β-NGF, VEGF, IL-6, TNF-α, IL-18, TGF-β1, and fractalkine/CX3CL1 were not associated with HTLV-1 PVL or the speed of HAM/TSP progression, which limits their use as biomarkers for HAM/TSP. Previously, we demonstrated that CSF levels of neopterin and CXCL10 can strongly predict the speed of HAM/TSP progression, with these factors better reflecting the disease activity than biomarkers of neuronal death, such as NfL and pNfH [11]. Despite the limitations of the study, particularly the sample size, the data we produced on the reduced CSF concentration of TGF-β1 in HTLV-1 infection and increased IL-18 in the CSF of HAM/TSP patients with active neuroinflammation indicate that unbalanced suppressive responses and inflammasome-mediated mechanisms in the CNS might contribute to HAM/TSP development. This points to new directions in the investigation of neurological diseases associated with HTLV-1 infection. In future work, the role of microglial cells and inflammasome responses in the HAM/TSP pathogenesis needs to be addressed.

## Figures and Tables

**Table 1:** Characteristics of the study population.

Characteristics	HTLV-1 AC (*n* = 13)	HAM/TSP (*n* = 20)	Control (*n* = 9)	*p*-Value ^f^
**Age (years)** ^a^	62.15 ± 10.21	54.75 ± 13.46	59.46 ± 20.65	0.345
**Sex** ^b^				
Male	6 (14.3%)	8 (19.0%)	4 (9.5%)	0.935
Female	7 (16.7%)	12 (28.6%)	5 (11.9%)	
**Time of infection (years)** ^c,d^	16.98 [IQR 16.47–21.71]	unknown	n.a.	n.a
**Time of disease (years)** ^a,e^	n.a.	12.94 ± 8.07	n.a.	n.a.
**Disability scale**				
Mild (1–10 points)	0	13 (65.0%)	n.a.	n.a.
Moderate (11–21 points)	0	5 (25.0%)	n.a.	n.a.
Severe (≥22 points)	0	2 (10.0%)	n.a.	n.a.
**CSF analysis**				
Total proteins (mg/dL) ^a^	48.25 ± 13.06	44.08 ± 12.86	33.00 ± 8.57	0.020
Frequency of elevated total proteins ^b^	53.8% (7/13)	30% (6/20)	0% (0/9)	0.027
Cell counts (cells/mm^3^) ^d^	1.0 [IQR 1–2]	4.0 [IQR 1.5–7.5]	1.0 [IQR 1–1]	<0.001
**Proviral load in PBMCs (%)** ^a^	4.50 ± 3.64	8.46 ± 6.37	n.a.	0.049

Notes: AC, asymptomatic HTLV-1 carrier; HAM/TSP, HTLV-1-associated myelopathy/tropical spastic paraparesis; n.a., non-applicable; IQR, interquartile range (25–75%). ^a^ Values are shown as the mean ± standard deviation. The statistical analysis was performed with an ANOVA test with post hoc Bonferroni correction. ^b^ Differences in the frequency between groups were evaluated with the chi-squared test. ^c^ The time of infection (in years) was defined as the interval between the laboratory diagnosis and sample collection. ^d^ Values are shown as the median and IQR. The statistical analysis was performed using the Kruskal–Wallis test with Dunn’s correction. ^e^ The time of disease (in years) was defined as the interval between the HAM/TSP diagnosis (pyramidal syndrome) and sample collection. ^f^ Results with a *p*-value < 0.05 were considered significant.

## Data Availability

The data presented in this study are available on request from the corresponding author.
